# Attitudes towards cosmetic surgery among women in China and the Netherlands

**DOI:** 10.1371/journal.pone.0267451

**Published:** 2022-04-21

**Authors:** Yi Wu, Jessica M. Alleva, Nick J. Broers, Sandra Mulkens

**Affiliations:** 1 Department of Clinical Psychological Science, Faculty of Psychology and Neuroscience, Maastricht University, Maastricht, The Netherlands; 2 Department of Methodology and Statistics, Faculty of Psychology and Neuroscience, Maastricht University, Maastricht, The Netherlands; 3 Department of Psychiatry and Neuropsychology, Faculty of Health, Medicine, and Life Sciences; School for Mental Health and Neuroscience, Maastricht University, Maastricht, The Netherlands; Fiji National University, FIJI

## Abstract

Around the world, an increasing number of people, predominantly women, are choosing to undergo cosmetic surgery—despite the associated health risks. This study aimed to promote a better cross-cultural understanding of the correlates and predictors of favorable attitudes toward cosmetic surgery among women in China (an Eastern country where cosmetic surgery is increasing most rapidly) and the Netherlands (a Western country). Questionnaire data were obtained from 763 adult women; 245 were Chinese women in China (*M*_*age*_ = 29.71), 265 were Chinese women in the Netherlands (*M*_*age*_ = 25.81), and 253 were Dutch women (*M*_*age*_ = 29.22). Facial appearance concerns and materialistic belief were significant predictors of favorable attitudes towards cosmetic surgery for all three cultural groups. Body appreciation was a significant positive predictor among Chinese women in both China and the Netherlands, whereas age and beauty-ideal internalization were significant positive predictors only among Chinese women in China. The findings and their implications are discussed with respect to the characteristics of Chinese culture that could explain the identified differences between Chinese and Dutch women.

## Introduction

Cosmetic surgery involves the ‘improvement’ of the physical appearance of body parts via elective surgical (e.g., liposuction) and non-surgical (e.g., filler treatment) procedures [1, 2]. It is one of the fastest-growing medical practices in the world, and it has become increasingly acceptable as the most effective means to modify appearance (e.g., [3]). As reported by the International Society for Aesthetic Plastic Surgery (ISAPS) in 2020, from 2015–2019, the worldwide number of cosmetic procedures increased from 20.1 million to 25 million [4]. Like all medical practices, cosmetic surgery is related with numerous potential undesirable consequences, such as cardiac complications, nerve damage, and infection [5–7]. Though a certain amount of evidence has supported that cosmetic surgery could produce psychosocial benefits [8–10], there were also studies showing a post-operative development of psychological problems (e.g., [11, 12]). Hence, cosmetic surgery seekers should cautiously and thoroughly consider and weigh the potential health risks that are not justifiable on health grounds but will be merely taken for aesthetic reasons; however, this is rarely observed among cosmetic surgery patients [13].

Given the unceasingly growing number of cosmetic procedures and their potential adverse consequences, a greater understanding of the correlates of favorable attitudes towards cosmetic surgery is needed. The present study focused on understanding these relationships among women, specifically. Globally, female physical attractiveness is considered a desirable trait [14], and women are viewed and evaluated based predominantly on their physical appearance, to a much greater extent than men are [15]. This mirrors global statistics showing that the majority (~90%) of cosmetic surgery patients are women throughout years (e.g., [4, 16]).

### Cross-cultural comparison between China and the Netherlands

This study also aimed to investigate the role of cultural context in women’s attitudes towards cosmetic surgery. Specifically, the Chinese cultural context was compared to the Dutch cultural context. In recent years, the Chinese cosmetic surgery market has been expanding six times faster than the global average [17]; China was ranked the third, after the US and Brazil, with respect to cosmetic surgery market in 2011 [18] and topped the world in 2018 [19, 20]. Meanwhile, as other countries, the main body of cosmetic surgery seekers are women in China [21]. To alert, the Chinese cosmetic surgery market is being extremely under-regulated, manifested in the existence of a great number of unlicensed surgeons and unqualified settings [2]. Nevertheless, several facets of Chinese culture and the social development process of China might help to understand Chinese women’s demand for cosmetic surgery. Under the profound influence of Confucius teachings, Chinese society is seen as more individualistic and male-oriented; women are taught to subject to male roles in family and social lives, and they are considered as “possessions” to their fathers and husbands [22, 23]. In line with that, female physical attractiveness is seen as a key determinant of Chinese femininity, particularly concerning its role in men’s sexual pleasure, as asserted in Taoism [24]. Socially, in parallel with the social driving force of China’s domestic beauty economy, which relates female beauty with economy [25], Chinese women are frightened to be eliminated in the fast-changing society during the modernization process and believe appearance improvement can somewhat buffer against their fear [26] because physically attractive women often fare better across various life domains (e.g., career, relationships; [27]).

The first thing that caught our research attention to compare Chinese women’s and Western women’s attitudes towards cosmetic surgery was the age difference between Chinese and Western cosmetic surgery patients. That Chinese cosmetic surgery patients are predominantly adolescent girls and young women, who are still in physical and psychological maturation and may not be fully capable to understand and cope with potential surgical risks (e.g., [28]). This is in marked contrast with Western countries, where cosmetic surgery patients are predominantly middle-aged and older women [29]. Among Western countries, the Netherlands was chosen as a country of interest for cross-cultural comparison based on the following considerations. First, though the idealized beauty in the Netherlands is similar to that of other Western countries (e.g., the U.S., the UK; [30]), and undergoing cosmetic surgery becomes increasingly common [31], there is very little research on cosmetic surgery among Dutch women. Socio-culturally speaking, Dutch society is commonly seen as more individualistic, and values the issue of gender equity more among countries [32, 33], which is in sharp contrast with Chinese culture and society. To demonstrate in a more direct way, the Global Gender Gap report has shown that the Netherlands was ranked 27^th^ while China was ranked 103^rd^ with respect to gender equity [34]. The noteworthy difference between women’s experiences in Chinese and Dutch society further made us interested in exploring whether Chinese and Dutch women would be motivated by different factors to undergo cosmetic surgery.

### Correlates of women’s attitudes towards cosmetic surgery

In addition to the advancement and improved accessibility of cosmetic surgery technology [35] as well as the availability of lower costs [36], other factors may also impact women’s attitudes towards cosmetic surgery. First of all, Western women who are *dissatisfied with their physical appearance* are motivated to carry out health-damaging behaviors (e.g., disordered eating; [37]) to improve their physical appearance; with the development of medical technology, cosmetic surgery becomes a means to improve appearance satisfaction [38]. Compared with being dissatisfied with the overall body appearance, being dissatisfied with particular appearance features seems to be a stronger and more consistent correlate of cosmetic surgery consideration [39, 40]. Most recent Chinese and Western findings suggest that the pursuit of cosmetic surgery may be more strongly correlated with *facial appearance dissatisfaction* (e.g., [27, 41, 42]), with a year-by-year increase in global facial cosmetic surgery being observed [4]. Further, with the global spread of consumerism and *materialism*, the importance of possessing materialistic wealth in determining life satisfaction and success has been overemphasized in both Eastern and Western cultures [43, 44]. Meanwhile, given that physical perfection and material success are tightly bound together and seen as two prominent symbols of an ideal feminine identity [36, 45], women who endorse materialistic values are more inclined to pursue a ‘perfect’ body and to *capitalize sexual attractiveness* (i.e., to regard sexual attractiveness as the most valuable asset that could be traded for positive life outcomes; [46]). Excessive media exposure to beauty ideals [47] paired with rewarding life events [48] further socializes women to believe that an idealized physical appearance is achievable and leads to a better life [36]. Cross-cultural research has shown that materialism and capitalization of sexual attractiveness are correlates of willingness to undergo cosmetic surgery among women, including adolescent girls (e.g., [49–52]).

As posited by *objectification theory* [53], living in a cultural context where women’s physical appearance is considered the defining feature of their worth can socialize women to engage in *self-objectification*, *or body surveillance*, whereby they come to value the self as a whole, based predominantly on how they look, from a third-person observer perspective. Women who objectify themselves are further vulnerable to *internalize the sociocultural beauty ideal* and compare their physical appearance against it [54]. Following that, the perceived gap between actual and idealized beauty could result in body image disturbance, a relationship which has been supported among Western (e.g., [55]) and Chinese women (e.g., [56]). Cosmetic surgery is widely acknowledged as the most ‘effective’ way to attain a ‘perfect’ physical appearance (e.g., [52]). Both Chinese and Western women have expressed willingness to weaken or eliminate psychological disturbances induced by self-surveillance and beauty-ideal internalization via cosmetic surgery (e.g., [7, 27, 41, 51]).

In addition to broader sociocultural influences, women’s more immediate social environment can also impact their attitudes towards cosmetic surgery. First, Chinese and Western studies have shown that people who have *appearance-related teasing experience*, particularly in childhood or adolescence, are more motivated to consider cosmetic surgery [57, 58]. Following this, female cosmetic surgery patients reported that they felt healed from the traumatic after-effects of the teasing experience due to becoming more confident with their physical appearance post-surgery [59]. Meanwhile, a more recent qualitative study reported that Chinese participants who underwent cosmetic surgery described cosmetic surgery helped them to say goodbye to the old self they did not like and made them feel ‘reborn’ postoperatively [58].

This study also considered facets related to age and their relationship with attitudes towards cosmetic surgery. Across cultures, cosmetic surgery is recognized as a means to remain youthful looking (underlined in feminine ideals; [60]) and to positively affect one’s career and relationship prospects [61]. *Ageing anxiety* (i.e., worries about the ageing process and anticipation of losses; [62]), especially with respect to physical appearance, is proposed to intensify the desire for cosmetic surgery [63]. This mirrors the fact that middle-aged women who experience the strongest ageing anxiety are more likely to be dissatisfied with their body and to consider having cosmetic surgery as a solution [37]. In line with this, *age* is also positively correlated with favorable attitudes toward cosmetic surgery among Western women (e.g., [64]). Statistically, Western female cosmetic surgery patients are mainly between 33–55 years of age [37], and 76% of cosmetic surgeries are performed on women above 40 years old in the U.S. [29]. In contrast, Chinese women may generally show favorable attitudes toward cosmetic surgery regardless of age: Although Chinese cosmetic surgery patients are mainly adolescent girls and young women [51], older women are also motivated to pursue cosmetic surgery to attain a more youthful appearance [60].

Facets of *positive body image*—“love and acceptance of one’s body (including aspects inconsistent with societally-prescribed ideals) and appreciation of its uniqueness and the functions it performs” [65 p. 168]—may also be associated with attitudes towards cosmetic surgery. First, people with higher *body appreciation*—a key facet of positive body image defined as the “unconditional approval and respect for the body” [66 p. 486]—are less inclined to internalize beauty ideals and to pursue cosmetic surgery for attaining these ideals [67]. Indeed, attitudes toward cosmetic surgery and body appreciation were found to be inversely correlated in prior studies among Western and non-Western (South Korean) women [68, 69]. However, two recent Chinese studies showed contradictory findings. A quantitative study by Wu and colleagues [70] did not find a significant relationship between acceptance towards cosmetic surgery and body appreciation. Following that, a qualitative exploration of the role of body appreciation in cosmetic surgery consideration among Chinese and Dutch women revealed a potential cross-cultural difference in conceptualizing the term “body appreciation” [58]. Namely, Dutch women’s conceptualization was in line with the formal definition presented in the (Western) body image literature [71], whereas Chinese women’s conceptualization tended to equate body appreciation with self-centeredness and appearance investment, with the element of unconditional acceptance being overlooked [58]. Another important facet of positive body image, *functionality appreciation*—an appreciation for what the body is capable of doing [72]—has been inversely associated with favorable attitudes towards cosmetic surgery among women in the U.S. When people appreciate and respect what their bodies can do, they might be more likely to avoid any procedures that could harm their bodies, such as cosmetic surgery [72]. To our knowledge, both body appreciation and functionality appreciation have not been investigated among Chinese and Dutch women, in relation to their attitudes toward cosmetic surgery.

### The present research

This study aimed to provide a better cross-cultural understanding of the correlates of attitudes toward cosmetic surgery among Chinese and Dutch women, living in their respective countries. To provide a more nuanced understanding of the potential impact of cultural factors on attitudes towards cosmetic surgery, we also included a group of Chinese women living in the Netherlands. Many of the associations under investigation have not been tested among Chinese women (i.e., appearance-related teasing experience, appearance-related ageing anxiety, and functionality appreciation) and none of these associations have been tested among Dutch women, until now. Potential differences in these associations between Chinese women and Dutch women have never been compared, despite the potential to provide meaningful information about cross-cultural differences in attitudes toward cosmetic surgery.

Based on the previous considerations, we expected attitudes towards cosmetic surgery to be positively associated with age, facial appearance concerns, materialistic belief, capitalization of sexual attractiveness, body surveillance, beauty-ideal internalization, appearance-related teasing experience, and ageing anxiety, and to be inversely associated with appearance evaluation and functionality appreciation among both Chinese and Dutch women. Concerning body appreciation, we expected it to be inversely associated with attitudes towards cosmetic surgery among Dutch women; meanwhile, we aimed to investigate this association in an exploratory manner considering the aforementioned two Chinese studies implying no or positive role of body appreciation in cosmetic surgery consideration.

Last, in addition to investigating the separate relationships between attitudes towards cosmetic surgery and the potential factors described above, we also investigated the *unique* contributions of each potential factor to attitudes towards cosmetic surgery, within each cultural group. In this way, we hoped to identify the strongest predictors of attitudes towards cosmetic surgery for Chinese women and Dutch women, after controlling for other relevant predictors. For this stage, we did not make specific hypotheses; however, we expected to see cross-cultural differences to have a better understanding of the influence of culture in cosmetic surgery context.

## Method

### Participants

After excluding the participants who indicated that they were not Chinese or Dutch, the final sample consisted of 763 women, who were Chinese women living in China (*n* = 245; 18–51 years), Chinese women living in the Netherlands (*n* = 265; 18–60 years), and Dutch women living in the Netherlands (*n* = 253; 18–71 years). Participants’ demographic characteristics are presented in Table 1. Of note, Chinese women in the Netherlands were significantly younger than the participants in the other two cultural groups (*p*s < .001), whereas no significant age difference was detected between Chinese participants in China and Dutch participants.

**Table 1:** Demographic characteristics.

	Chinese women in China (*N* = 245)		Chinese women in the Netherlands (*N* = 265)		Dutch women in the Netherlands (*N* = 253)	
Demographic Characteristics	*M*	*SD*	*M*	*SD*	*M*	*SD*
**Age**	29.71	6.94	25.81	5.21	29.22	13.50
**Body mass index**	20.93	3.66	20.52	3.24	23.78	4.45
	***N***	**%**	***N***	**%**	***N***	**%**
**Personal cosmetic surgery experience**						
**Yes**	39	15.9	18	7.6	20	8.8
**No**	206	84.1	219	92.4	208	91.2
**Employment status**						
**Employed**	196	80.0	66	27.3	88	38.1
**Unemployed (including students)**	49	20.0	176	72.7	143	61.9
**Relationship status**						
**In a relationship**	175	71.4	120	49.6	141	61.0
**Not in a relationship**	70	28.6	122	50.4	90	39.0
**Education level**						
**Middle school**	1	.4	0	0	6	2.6
**High school**	8	3.3	7	2.9	51	22.4
**Bachelor**	196	81.3	70	29.2	95	41.7
**Master**	30	12.4	113	47.1	67	29.4
**PhD**	6	2.5	50	20.8	9	3.9
**Vicarious cosmetic surgery Experience**						
**0**	19	7.8	36	14.9	60	26
**1–5**	174	71	176	72.7	143	61.9
**6–10**	35	14.3	20	8.3	22	9.5
**More than 10**	17	6.9	10	4.1	6	2.6
**Length of stay in the Netherlands** *						
**1 year**			156	60.2		
**2–3 years**			65	25.1		
**4–5 years**			25	9.7		
**More than 5 years**			13	5		

Note.

* Length of stay in the Netherlands was answered by the Chinese women in the Netherlands only.

### Measurements

Participants’ scores on the measurements, described below, are presented in Table 2, along with the Cronbach’s alphas (all of which demonstrated acceptable internal reliability).

**Table 2:** Descriptive statistics for measured variables.

	Chinese women in China^a^		Chinese women in the Netherlands^b^		Dutch women in the Netherlands^c^					
Variables	Cronbach’s *ɑ*	*M (SD)*	Cronbach’s *ɑ*	*M (SD)*	Cronbach’s *ɑ*	*M (SD)*	*F*	*df*_*between*_	*df*_*within*_	*p*
**Attitudes Towards Cosmetic Surgery**	.96	4.71 (1.31)^b,c^	.93	4.21 (1.15)^a,c^	.94	3.73 (1.30)^a,b^	37.849	2	760	< .001
**Appearance Evaluation**	.84	3.25 (.71)^c^	.80	3.39 (.65)	.86	3.44 (.73)^a^	5.056	2	760	.007
**Facial Appearance Concerns**	.87	2.37 (.65) ^b,c^	.88	2.19 (.72) ^a,c^	.90	1.84 (.72) ^a,b^	37.050	2	760	< .001
**Body Surveillance**	.84	4.25 (1.05)^c^	.78	4.07 (.98)^c^	.82	4.52 (1.08)^a,b^	12.262	2	760	< .001
**Body Appreciation**	.88	3.56 (.66)^b^	.92	3.77 (.69)^a^	.95	3.65 (.80)	5.919	2	760	.003
**Functionality Appreciation**	.72	4.04 (.48)^b^	.88	4.19 (.53)^a^	.89	4.15 (.56)	5.335	2	727	.005
**Materialistic Belief**	.89	3.32 (.63) ^b,c^	.81	3.11 (.55) ^a,c^	.85	2.54 (.67) ^a,b^	108.225	2	754	< .001
**Beauty-Ideal Internalization**	.76	3.35 (.43)	.77	3.26 (.51)	.85	3.22 (.64)	3.937	2	732	.020
**Appearance-Related Ageing Anxiety**	.79	15.49 (4.35) ^b,c^	.73	13.44 (4.20) ^a,c^	.74	11.13 (4.44) ^a,b^	61.064	2	729	< .001
**Capitalization of Sexual Attractiveness**	.90	4.33 (1.35)	.87	4.31 (1.26)	.90	4.40 (1.37)	.269	2	713	.764
**Appearance-Related Teasing**	.87	10.18 (3.97)	.87	9.87 (4.01)	.78	9.54 (3.53)	1.644	2	712	.194

*Note*. Superscript notes (a–d) indicate which groups significantly differ from each other (*p* < .001).

#### Demographic characteristics

Participants reported their age, highest education level, employment status, relationship status, previous experience of receiving cosmetic surgery (yes/no), vicarious cosmetic surgery experience (“How many people do you know who have undergone cosmetic surgery?”), and height and weight. Of note, although data concerning weight and height were collected to calculate body mass index (BMI) for descriptive purposes, due to a computer error these data could not be used. Namely, participants were required to enter only a number for their weight, and we were therefore unable to determine whether participants had reported their weight in kg (common in the Netherlands) or 500g (common in China).

#### Attitudes towards cosmetic surgery

The 15-item Acceptance of Cosmetic Surgery Scale (ACSS; [64]) was used to measure participants’ attitudes toward cosmetic surgery from three perspectives (five items each), namely (1) Intrapersonal (e.g., “If cosmetic surgery can make someone happier with the way they look, then they should try it”), (2) Social (e.g., “I would seriously consider having cosmetic surgery if my partner thought it was a good idea”), and (3) Consider (e.g., “In the future, I could end up having some kind of cosmetic surgery;” [64]). This scale has also been translated in Chinese and validated among Chinese adults [70]. Items are rated on a seven-point Likert scale, from 1 = *strongly disagree* to 7 = *strongly agree*; higher mean scores across items indicate more positive attitudes toward cosmetic surgery. Satisfactory reliability and validity were supported in both Western and Chinese studies (e.g., [64, 70, 73]).

#### Appearance evaluation

The seven-item Appearance Evaluation Subscale of the Multidimensional Body-Self Relations Questionnaire (MBSRQ; [74, 75]) was used to measure overall appearance evaluation. Items (e.g., “I like the way my clothes fit me”) are rated on a five-point Likert scale, from 1 = *definitely disagree* to 5 = *definitely agree*; higher mean scores across items indicate more positive appearance evaluation. Adequate psychometrics properties were evidenced in Western and Eastern studies (e.g., [76–78]).

#### Facial appearance concerns

The 11-item Facial Appearance Concerns Subscale of the Negative Physical Self Scale (NPS; [79]) was used to measure participants’ thoughts and feelings toward their facial appearance (e.g., “I am depressed about how my face looks”). Items are rated on a five-point Likert scale, from 1 = *not like me at all* to 5 = *always like me*; higher mean scores across items indicate higher levels of facial appearance concerns. The NPS was initially developed among Chinese adults, in Chinese; simultaneously, the English translation of it was provided [79]. Satisfactory reliability and validity were confirmed among Chinese women (e.g., [41, 70, 79]).

#### Materialistic belief

The 15-item Materialistic Value Scale [80] was used to assess how participants value materialistic things from three dimensions: success, centrality, and happiness. All items (e.g., “Buying things gives me a lot of pleasure”) are rated on a five-point Likert Scale, from 1 = *strongly disagree* to 5 = *strongly agree*; higher mean scores across items indicate higher materialistic values. Reliability was supported in the literature, among Western and Chinese women (e.g., [81, 82]).

#### Capitalization of sexual attractiveness

The five-item Capitalization of Sexual Attractiveness Scale [46] was used to measure participants’ tendency to capitalize on female sexual attractiveness. Items (e.g., “Sexual attractiveness is a profitable resource”) are rated on a seven-point Likert scale, from 1 = *totally disagree* to 7 = *totally agree*; higher mean scores across items indicate a greater tendency to capitalize sexual attractiveness. Reliability was supported among Chinese women [46].

#### Body surveillance

The eight-item Body Surveillance Subscale of the Objectified Body Consciousness Scale (OBCS; [83]) was used to assess participants’ levels of body surveillance (e.g., “I often worry about whether the clothes I am wearing make me look good”). Items are rated on a seven-point Likert scale, from 1 = *strongly disagree* to 7 = *strongly agree*; higher mean scores across items indicate higher levels of body surveillance. Adequate psychometrics properties were evidenced among Western and Chinese women (e.g., [46, 84, 85]).

#### Beauty-ideal internalization

The 15-item Internalization of Beauty Ideals Subscale of the Sociocultural Attitudes Towards Appearance Questionnaire-4-Revised (SATAQ-4R; [86]) was used to assess beauty-ideal internalization. This subscale captures three dimensions: Internalization of (1) thinness, (2) muscularity, and (3) general attractiveness. Items (e.g., “I think a lot about looking thin”) are rated on a five-point Likert scale, from 1 = *definitely disagree* to 5 = *definitely agree*; higher mean scores across items indicate a higher tendency to internalize societal beauty ideals. The SATAQ-4R has been translated in Chinese and validated among Chinese adults [70]. Adequate psychometric properties were evidenced among Western and Chinese women (e.g., [70, 87]).

#### Appearance-related teasing

This six-item Appearance-Related Teasing Subscale of the Perception of Teasing Scale (POTS; [88]) was used to assess participants’ experience of appearance-related teasing, with respect to Frequency (e.g., “People pointed at you because of your appearance”) and Effect (i.e., “How upset were you after being teased”). In this study, only the responses to the Frequency items were analyzed. The Frequency items are rated on a five-point Likert scale, from 1 = *never* to 5 = *very often*; higher sum scores, ranging from 6 to 30, indicate higher frequency of being teased. Satisfactory reliability was supported among Western women (e.g., [88, 89]).

#### Appearance-related ageing anxiety

The Physical Appearance Subscale of the Anxiety about Ageing Scale (AAS; [62]) contains five items, and measures ageing-related anxiety. Items (e.g., “I have never dreaded looking old”) are rated on a five-point Likert Scale, from 1 = *strongly agree* to 5 = *strongly disagree*; higher sum scores, ranging from 5 to 25, indicate higher levels of appearance-related ageing anxiety. Satisfactory reliability was supported among Western women (e.g., [90, 91]).

#### Body appreciation

The 10-item Body Appreciation Scale-2 [71] was used to assess body appreciation, and it has been translated and validated in Chinese [92]. Items (e.g., “I feel good about my body”) are rated on a five-point Likert scale, from 1 = *strongly disagree* to 5 = *strongly agree*; higher mean scores across items indicate higher levels of body appreciation. Satisfactory reliability and validity were well-supported in both Western and Chinese literature (e.g., [70, 71, 92]).

#### Functionality appreciation

The seven-item Functionality Appreciation Scale (FAS; [72]) was used to assess functionality appreciation (e.g., “I acknowledge and appreciate when my body feels good and/or relaxed”). Items are rated on a five-point Likert scale, from 1 = *strongly disagree* to 5 = *strongly agree*; higher mean scores across items indicate higher levels of functionality appreciation. Satisfactory reliability was supported among U.S. women [72].

### Procedure

The research was approved by the Ethics Review Committee Psychology and Neuroscience at Maastricht University (ethics approval code Master_206_33_03_2019_A1), and was preregistered on AsPredicted (protocol #25692, see https://aspredicted.org/ve5bu.pdf [PROTOCOL DOI]). Of note, after preregistration, one change was made to the analysis plan. Namely, although we had administered two additional subscales—the Body Control Subscale of the OBCS and the Appearance Pressures Subscale of the SATAQ-4R, we did not prepare or analyze these data. Prior to data preparation, we consulted with our department’s statistician (NJB), who advised us to prioritize 11 variables (aside from the ACSS) to strengthen our analytical plan. These two subscales were removed from the dataset as they were deemed less important (theoretically) in comparison to the remaining 11 variables and the Body Control Subscale of the OBC seems to show a week conceptual basis and generally poor psychometrics in the literature.

The Chinese participants in China were recruited via Wen Juan Xing (i.e., a widely used online survey platform), and the Chinese and Dutch participants in the Netherlands were recruited via Facebook, flyers, the university’s online research system, and snowball sampling. Inclusion criteria were identifying as female and being ≥18 years old; also, for participants in the Netherlands, they were required to be proficient in English; there was no exclusion criterion. The questionnaires for Chinese participants in China were in Chinese, whereas for Chinese and Dutch participants in the Netherlands they were in English. The standard back-translation technique [93] was employed for translating questionnaires that were not available in Chinese when the study began (i.e., FAS, POTS, Appearance Evaluation Subscale, Physical Appearance Subscale). The first author, a native Chinese speaker, first translated these questionnaires into Chinese. Two Chinese-English bilingual scholars, unaffiliated with the study, translated the questionnaires from Chinese back into English. The back-translated questionnaires were compared with the original ones to correct any detected discrepancies, all concerned wording, and ensure the Chinese translations precisely captured the meaning of the original English ones.

All data were collected anonymously via the online research platforms Wen Juan Xing (Chinese participants in China) and Qualtrics (participants in the Netherlands). Data collection took place between March and August, 2019. Participants first read an information letter about the purpose of the study, describing that they would complete questionnaires to assess their attitudes towards cosmetic surgery. Participants then signed an electronic informed consent form and proceeded to the questionnaires. The average time for completion was 24 minutes. As compensation, participants could choose between research credits (if they were students at Maastricht University) and a chance to win a raffle.

### Statistical analyses

All statistical analyses were conducted using SPSS 25. Data were screened for normality, outliers, and missing data. A series of ANOVAs with post-hoc analyses were carried out to compare the means on the continuous variables across the three cultural groups. To control for multiple testing without overly compromising on statistical power, we consistently used an alpha of 0.01 for all omnibus tests.

Within each cultural group, bivariate correlations between the ACSS scores and the scores on the 11 additional questionnaires were examined. Subsequently and separately for each cultural group, backward multiple regression analyses were carried out to identify the optimal model for explaining the ACSS scores on the basis of the 11 variables. To minimize chance capitalization, each cultural sample was randomly divided into two subsamples (i.e., S1 and S2). S1 was firstly examined exploratively with the *backwards* analysis and predictors with *p* > .10 were sequentially removed until a model resulted with only significant predictors. After that, S2 was used as a validation sample for a confirmatory analysis. All predictors that were found to significantly predict ACSS scores in S1 were entered in this model, and the *R*^2^ value was registered. Next, the aforementioned procedure was repeated but in a reverse manner: S2 was tested by backwards analysis (starting with all 11 predictors at the initial step) and then S1 was used as the validation sample for providing a confirmatory analysis of the end result found in S2. Again, the *R*^2^ value for the confirmatory analysis was registered. This procedure yielded two end models, and the model with the higher *R*^2^ value was chosen as the best one for describing that cultural group.

In the final step, we integrated our findings by formulating a regression model for all three cultural groups combined. All predictors that proved significant for at least one of the cultural groups were entered in this integrated model. In addition, product terms were included to permit the modeling of moderating effects. A hierarchical regression analysis was carried out with an initial model containing only main effects of all predictors plus dummy variables for the identification of cultural group, a second model additionally contained product terms to model predictors by cultural group interactions for those predictors that had been found to differ across cultural groups in the explorative stage, and lastly a third model also included interactions with cultural groups for those predictors that did not behave very differently across cultural groups. We expected all three models to be significant, with the second model to show a significant improvement of the first, but the third not showing a significant improvement of the second. To enhance the interpretability, all of the predictors that appeared in the hierarchical regression models were transformed into centered scales. Membership of cultural groups was identified by creating dummy variables for the two Chinese groups, leaving the Dutch group as the reference group in the model (for further details on this statistical approach, see [94]).

## Results

### Cross-cultural comparisons of questionnaire scores

Descriptive statistics for all the examined variables are presented in Table 2. With the exception of beauty-ideal internalization, capitalization of sexual attractiveness, and appearance-related teasing, the scores were all significantly different between cultural groups.

Post-hoc comparisons indicated that for the scales measuring favorable attitudes towards cosmetic surgery, facial appearance concerns, materialistic belief, and appearance-related ageing anxiety, Chinese participants in China scored significantly higher than Chinese participants in the Netherlands; also, they both scored significantly higher than Dutch participants. For the scales measuring body appreciation and functionality appreciation, Chinese participants in China scored significantly lower than Chinese participants in the Netherlands; no difference was detected between the Dutch participants’ scores and those of either of the Chinese groups of women. For the scales measuring appearance evaluation and body surveillance, Chinese participants in China scored significantly lower than Dutch participants; Chinese participants in the Netherlands also scored lower than Dutch participants regarding body surveillance.

### Correlations between ACSS scores and additional variables

The within-group correlations between favorable attitudes towards cosmetic surgery and its potential predictors are shown in Tables 3 and 4. Favorable attitudes towards cosmetic surgery were correlated with facial appearance concerns, body surveillance, materialistic belief, beauty-ideal internalization, appearance-related ageing anxiety, capitalization of sexual attractiveness, and appearance-related teasing experience within each of the three cultural groups. Some of the predictors correlated with favorable attitudes towards cosmetic surgery only within one or a subset of cultural groups. Specifically, favorable attitudes towards cosmetic surgery were inversely correlated with appearance evaluation and body appreciation among Chinese participants living in the Netherlands and Dutch participants only, as well as with functionality appreciation among Dutch participants only. Age was not correlated with favorable attitudes towards cosmetic surgery in any of the cultural groups.

**Table 3:** Correlations between variables among Chinese women in China and in the Netherlands.

**Variables**	**1**	**2**	**3**	**4**	**5**	**6**	**7**	**8**	**9**	**10**	**11**	**12**
**1. Attitudes Towards Cosmetic Surgery**	-	- .15*	.48***	.35***	- .15*	- .08	.38***	.33***	.22***	.28***	.17**	- .04
**2. Appearance Evaluation**	- .08	-	- .50***	- .24***	.62***	.39***	- .07	- .12	- .04	- .13	- .29***	.04
**3. Facial Appearance Concerns**	.49***	- .55***	-	.41***	- .55***	- .30***	.31***	.30***	.17**	.25***	.35***	- .16**
**4. Body Surveillance**	.42***	- .15*	.34***	-	- .36***	- .17**	.36***	.47***	.10	.33***	.18**	- .24***
**5. Body Appreciation**	- .08	.71***	- .52***	- .27***	-	.62***	- .11	- .16*	- .04	- .17**	- .29***	.16**
**6. Functionality Appreciation**	.09	.20**	- .14*	- .14*	.47***	-	- .05	- .04	- .08	.001	- .12	- .04
**7. Materialistic Belief**	.43***	- .21**	.35***	.57***	- .24***	.004	-	.40***	.20**	.22**	.08	- .31***
**8. Beauty-Ideal Internalization**	.52***	- .04	.28***	.39***	- .10	.02	.39***	-	.10	.26**	.07	- .14*
**9. Appearance-Related Ageing Anxiety**	.34***	- .22***	.36***	.52***	- .38	- .20**	.43***	.32***	-	.11	- .06	.02
**10. Capitalization of Sexual Attractiveness**	.32***	- .28***	.32***	.38***	- .26***	.03	.53***	.19**	.37***	-	.16*	- .10
**11. Appearance-Related Teasing**	.21**	- .42***	.59***	.17**	- .40	- .14	.23***	.11	.24***	.15*	-	- .04
**12. Age**	.08	- .004	.004	- .18**	.04	- .02	- .08	- .03	.02	- .07	- .03	-

*Note*. Correlations for Chinese women in China are below the diagonal; correlations for Chinese women in the Netherlands are above the diagonal;

*p < .05;

**p < .01;

***p < .001.

**Table 4:** Correlations between variables among Dutch women.

**Variables**	**1**	**2**	**3**	**4**	**5**	**6**	**7**	**8**	**9**	**10**	**11**	**12**
**1. Attitudes Towards Cosmetic Surgery**	-											
**2. Appearance Evaluation**	- .20**	-										
**3. Facial Appearance Concerns**	.49***	- .55***	-									
**4. Body Surveillance**	.37***	- .33***	.36***	-								
**5. Body Appreciation**	- .27***	.73***	- .62***	- .49***	-							
**6. Functionality Appreciation**	- .16*	.46***	- .35***	- .26***	.59***	-						
**7. Materialistic Belief**	.45***	- .10	.32***	.44***	- .24***	- .14*	-					
**8. Beauty-Ideal Internalization**	.27***	- .23***	.30***	.47***	- .28***	- .14*	.35***	-				
**9. Appearance-Related Ageing Anxiety**	.27***	- .01	.24***	.22**	- .19**	- .13	.28***	.25***	-			
**10. Capitalization of Sexual Attractiveness**	.23***	- .16*	.22**	.17*	- .17**	- .08	.25***	.15*	.05	-		
**11. Appearance-Related Teasing**	.22**	- .34***	.49***	.30***	- .49***	- .33***	.19**	.21**	.03	.06	-	
**12. Age**	- .12	.15*	- .21**	- .41***	.29***	.07	- .36***	- .34***	.02	- .09	- .25***	-

Note.

*p < .05;

**p < .01;

***p < .001.

### Multiple regression analyses to predict ACSS scores

The final models that best explained favorable attitudes towards cosmetic surgery for each cultural group, separately, are presented in Table 5. There were five significant predictors identified in the final model for Chinese participants in China: facial appearance concerns, body appreciation, materialistic belief, age, and beauty-ideal internalization. The three identified significant predictors in the final model for Chinese participants in the Netherlands were facial appearance concerns, body appreciation, and materialistic belief. The two identified significant predictors in the final model for Dutch participants were facial appearance concerns and materialistic belief. Of note, in each of the final models, all of the predictors *positively* predicted ACSS scores.

**Table 5:** Multiple regression models for all three cultural groups.

	Unstandardized coefficient		Standardized coefficient						
Predictors	B	SE	*β*	*t*	*p*	*R* ^*2*^	Adjusted *R*^*2*^	*F*	*p*
**Chinese in China**									
**Overall Model**						.465	.454	41.513	< .001
**(constant)**	- 4.438	.781		- 5.680	.000				
**Facial Appearance Concerns**	.881	.119	.436	7.414	.000				
**Body Appreciation**	.448	.112	.223	4.003	.000				
**Materialistic Belief**	.413	.112	.198	3.689	.000				
**Age**	.019	.009	.098	2.056	.041				
**Beauty-Ideal Internalization**	1.056	.159	.346	6.624	.000				
**Chinese in the Netherlands**									
**Overall Model**						.321	.313	40.457	< .001
**(Constant)**	- .086	.589		- .147	.884				
**Facial Appearance Concerns**	.806	.101	.513	7.947	.000				
**Body Appreciation**	.267	.101	.163	2.635	.009				
**Materialistic Belief**	.490	.113	.236	4.352	.000				
**Dutch in the Netherlands**									
**Overall Model**						.331	.325	61.234	< .001
**(Constant)**	.862	.282		3.061	.002				
**Facial Appearance Concerns**	.683	.098	.383	6.980	.000				
**Materialistic Belief**	.632	.107	.324	5.913	.000				

### Comparison of final models across cultural groups

Table 6 gives an overview of the *F* change tests pertaining to the hierarchical regression analysis. As predicted, including interaction terms involving facial appearance concerns and materialistic belief did not significantly improve the model. Also in line with our predictions, the second model proved to give the best description of the cross-cultural relationships between attitudes towards cosmetic surgery and cultural group, facial appearance concerns, body appreciation, materialistic belief, beauty-ideal internalization, age, and the cultural group interaction terms involving body appreciation, beauty-ideal internalization, and age. The two product terms related to the predicted moderation of age by cultural group proved to be nonsignificant and for this reason we have re-analyzed the final model with the exclusion of these two interaction terms.

**Table 6:** Hierarchical cross-cultural model comparison.

				Change statistics				
Step	*R*	*R* ^*2*^	*Adjusted R* ^*2*^	*R* ^*2*^ *change*	*F change*	*df1*	*df2*	*p*
**1** ^a^	.647	.418	.413	.418	74.661	7	727	< .001
**2** ^b^	.663	.440	.430	.022	4.654	6	721	< .001
**3** ^c^	.665	.442	.429	.002	.749	4	717	.559

Note.

^a^Facial Appearance Concerns, Body Appreciation, Materialistic Belief, Beauty-Ideal Internalization, Age, and indicator variables for identifying cultural group (with Dutch group as reference);

^b^Model 1 expanded with Interactions between cultural group and Body Appreciation, cultural group and Beauty-Ideal Internalization, and cultural group and Age;

^c^Model 2 expanded with interactions between cultural group and Facial Appearance concerns, and cultural group and Materialistic Belief.

Within the second model, the significant terms were main effects for facial appearance concerns, materialistic belief and age, as well as by cultural group interaction terms involving body appreciation and beauty-ideal internalization. The significant interactions revealed that body appreciation and beauty-ideal internalization made significantly higher positive contributions to attitudes towards cosmetic surgery among Chinese participants living in China relative to Dutch participants. Conversely, there was no indication of a significant difference between Chinese and Dutch women living in the Netherlands (Table 7).

**Table 7:** Regression estimates of model 2^a^.

	Unstandardized coefficients							
Variables^b^	B	SE	*β*	*t*	*p*	*R* ^*2*^	*F*	*p*
						.439	51.342	< .001
**(constant)**	4.184	.072		57.729	< .001			
**Facial Appearance Concerns**	.782	.067	.435	11.655	< .001			
**Body Appreciation**	.102	.093	.056	1.097	.273			
**Materialistic Belief-**	.523	.068	.277	7.658	< .001			
**Beauty-Ideal Internalization**	.193	.111	.078	1.743	.082			
**Age**	.009	.004	.062	2.014	< .05			
**ChNL**	- .086	.101	- .031	- .848	.397			
**ChCH**	.113	.106	.040	1.063	.288			
**Body Appreciation _ChNL**	.153	.125	.048	1.226	.221			
**Body Appreciation _ChCH**	.320	.130	.093	2.462	< .05			
**Beauty-Ideal Internalization _ChNL**	.054	.166	.012	.326	.745			
**Beauty-Ideal Internalization _ChCH**	.833	.186	.158	4.491	< .001			

Note.

^a^The cultural group by Age interaction proved to be non-significant and was removed.

^b^All continuous variables are expressed on centered scales. Binary variables are (0,1) indicator variables for identifying cultural group membership. Dutch group forms the reference group.

## Discussion

The global cosmetic surgery market has witnessed a drastic expansion in recent years, with China ranked first place [19, 20]; an increasing number of women seek cosmetic surgery for appearance ‘improvement’ [4, 7]. This study aimed to better understand attitudes towards cosmetic surgery among Chinese women—living in China and living in The Netherlands—and Dutch women. Below, we discuss the key findings, especially those related to predictors in different cultural groups, and implications that have emerged from our data.

### Levels of favorable attitudes towards cosmetic surgery and its potential correlates

First, examination of levels of favorable attitudes towards cosmetic surgery, and its potential correlates, across cultural groups yielded noteworthy findings. Chinese and Dutch women scored similarly on beauty-ideal internalization, capitalization of sexual attractiveness, and appearance-related teasing. However, Chinese women in both groups scored higher on favorable attitudes towards cosmetic surgery, facial appearance concerns, materialistic beliefs, and appearance-related ageing anxiety. These findings are consistent with the data showing that the Chinese cosmetic surgery market is expanding faster than the global average [21], facial features are emphasized in Chinese beauty ideals [36], Chinese people endorse the strongest materialistic beliefs in the world [95], and youthfulness is perceived to have marketing value [96]. Besides, Wu et al.’s [58] qualitative finding showed that married Chinese women were motivated to undergo anti-ageing cosmetic procedures to maintain an attractive youthful appearance in response to the insecurity of marriage stability; however, this motivation was not brought up by any Dutch participant. Of note, because Chinese women in the Netherlands scored significantly lower on these variables compared to Chinese women in China, the potential influence of culture on reducing the level of these variables could be inferred. This is noteworthy considering that most of the Chinese women in the Netherlands (95%) reported living there for four years or less (60% for one year or less). Further, people who go abroad are characterized by higher eagerness to understand other cultures [97] and greater openness relative to people who do not go abroad [98]. These characteristics may also facilitate the impact of cultural influence on Chinese women who move to the Netherlands.

Furthermore, scores on body appreciation and functionality appreciation, two key facets of positive body image [71], only differed between the two Chinese groups, with Chinese women in China scoring significantly lower than Chinese women in the Netherlands. Beyond that, Dutch women scored significantly higher on appearance evaluation compared to Chinese women in China. This finding supports other data showing that Chinese women had greater appearance dissatisfaction compared with the U.S. women, another Western sample [99], which might be explained by heightened media and social pressures with respect to appearance (e.g., [100]). In addition, lower levels of body surveillance among Chinese women in both groups compared to Dutch women could be partly explained by the fact that the items measuring body surveillance were more directed at women’s monitoring of their general appearance and clothing, which are less stressed in Chinese beauty ideals compared to facial appearance [41].

### Correlates of favorable attitudes towards cosmetic surgery

With respect to the correlates of favorable attitudes towards cosmetic surgery, most of our findings were in line with our hypotheses. That is, favorable attitudes towards cosmetic surgery were positively correlated with facial appearance concerns, body surveillance, beauty-ideal internalization, materialistic beliefs, capitalization of sexual attractiveness, ageing anxiety, and appearance-related teasing within all three cultural groups. Meanwhile, favorable attitudes towards cosmetic surgery were inversely correlated with functionality appreciation among Dutch women only. Given that cosmetic surgery has been normalized in China [2] and potential risks of cosmetic surgery are generally not disclosed on advertisements and during pre-surgical consultation, it could be that Chinese women are less aware of the risks of cosmetic surgery for their body functionality (e.g., numbness, nerve damage; [101]).

Attitudes towards cosmetic surgery were inversely correlated with appearance evaluation and body appreciation, only among Chinese women in the Netherlands and Dutch women. As described above, it could be that general appearance evaluation is not related to attitudes towards cosmetic surgery among women in China because facial appearance is viewed as more important than bodily appearance [36]. Also, women who are motivated to undergo cosmetic procedures are seen as being dissatisfied with particular appearance features that are aimed for cosmetic surgery; meanwhile, they could be satisfied with their overall body image [58]). With respect to body appreciation, though it was not a correlate (cf. [70]), it was found to be a significant positive predictor of attitudes towards cosmetic surgery among Chinese women in China, as discussed further below.

Of note, age was not correlated with favorable attitudes towards cosmetic surgery in any of the three cultural groups. This was consistent with the findings among South Korean [69] and Caucasian women [102]. Our findings imply that women might consider ‘improving’ their appearance, regardless of age, partly due to appearance-related pressures (e.g., employment, marriage security) that remain across the lifespan [3].

### Predictors of favorable attitudes towards cosmetic surgery

We further explored the unique contributions made by each predictor of attitudes towards cosmetic surgery. First, facial appearance concerns and materialistic beliefs were significant positive predictors of attitudes towards cosmetic surgery in all three cultural groups. This is consistent with figures showing that facial cosmetic procedures are most popular compared to other cosmetic procedures in China [2, 36, 96], and facial cosmetic procedures continue to increase in Western countries [4]. Potentially, the worldwide usage of ‘selfies’ and social media may also promote attention to facial features, especially those that are perceived as ‘flawed’ [103]. Concerning materialistic beliefs, with the global economic change and the emergence of consumerism [43], materialistic possessions are underlined in life satisfaction and life success [44]. Meanwhile, the instrumental value of physical beauty—e.g., having beautiful appearance can increase job competitiveness—is magnified in today’s society, particularly on social media (e.g., [20]). In consistency, our findings suggest that the positive association between physical attractiveness and materialistic success—often depicted together for commercialized purposes [48] and seen as two prominent symbols of an ideal feminine identity [36, 45]—influence both Chinese and Dutch women’s attitudes towards cosmetic surgery. To pursue materialistic success, women may be driven to achieve a ‘perfect’ appearance via cosmetic surgery [52].

With respect to age, our findings suggest that an increase in age could predict greater favorable attitudes towards cosmetic surgery among Chinese women in China. Interestingly, Chinese cosmetic surgery patients are predominantly adolescent girls and young adult women [51]. Therefore, despite endorsing greater favorable attitudes towards cosmetic surgery relative to younger Chinese women, older Chinese women might be less likely to undergo cosmetic surgery, which requires further understanding. To obtain higher social status, greater social resources [104, 105], and higher marriage security [2, 106], older Chinese women may be more motivated to consider cosmetic surgery to look younger and more attractive.

Beauty-ideal internalization was also found to be a positive predictor of attitudes towards cosmetic surgery among Chinese women in China. Being influenced by collectivistic culture, Chinese women in China are prone to seeking uniformity in physical appearance [107], internalizing social standards, and striving to meet appearance standards [32, 108]. These characteristics could explain how beauty-ideal internalization could predict favorable attitudes towards cosmetic surgery.

Conspicuously, age and beauty-ideal internalization were not significant predictors of attitudes towards cosmetic surgery among Chinese and Dutch women in the Netherlands; this might signify the influence of Dutch culture. First, compared to women in China, women in the Netherlands may be less subject to (explicit) age discrimination, for example via employers who require that potential job candidates be young and “attractive” as is common in China [2]. Second, being influenced by individualistic culture, people in Western countries tend to prioritize intrapersonal, rather than interpersonal, perspectives in decision-making [32, 109], which may explain why attitudes towards cosmetic surgery are not predicted by beauty-ideal internalization among women in the Netherlands. Beyond that, as found by Jackson et al. [110], Chinese undergraduate women developed greater appearance pressure after viewing physical appearance depictions in Chinese/Asian mass media compared to Western mass media. This implies that people are more likely to compare themselves to others with whom they are more similar [111]. Thus, Chinese women in the Netherlands who were exposed to mainly Western physical appearances in daily life and on media may have been less likely to internalize Western beauty ideals and thus, their attitudes towards cosmetic surgery may have been less likely to be predicted by beauty-ideal internalization.

Interestingly, body appreciation was found to predict greater favorable attitudes towards cosmetic surgery among Chinese women, both in China and in the Netherlands, which contrasts the literature showing an inverse correlation between them (e.g., [69]). These differences might be due to the fact that prior studies stopped at zero-correlation findings instead of proceeding to regression analyses, or due to cross-cultural differences in how body appreciation is conceptualized. Namely, in Western literature, body appreciation is characterized by unconditional body acceptance [71], and women whose bodies are unconditionally accepted and loved by influential others (e.g., family, friends) tend to have higher body appreciation [66, 112]. In contrast, Chinese people tend to believe love and acceptance are conditional, which may be related to Chinese authoritarian parenting style that is characterized by low warmth and high control (e.g., [113]), and by only appreciating their children after they have complied to social expectations and norms [114]. In addition, influenced by Confucian values, Chinese children are educated to honor their families with their achievements and superiority compared with others—otherwise, they will make their families ashamed [115, 116]. These characteristics have been supported by the recent qualitative finding reported by Wu and colleagues [58]; meanwhile, their research participants have described that Chinese women who love and appreciate their body also believe that they must continue to strive to be ‘better’ and ‘better’—even if that means using cosmetic surgery to improve their appearance.

### Cross-cultural differences on predictors

No significant difference was identified between Chinese women in the Netherlands and Dutch women with respect to the aforementioned significant predictors for all three cultural groups; this implies that attitudes towards cosmetic surgery and its predicting model could be shaped by sociocultural influence. Meanwhile, such influence did not require a long time to show its effect, because the majority of our Chinese participants had come to the Netherlands no more than four years ago, which might be facilitated by their openness to cross-cultural understanding [117]. The first cross-cultural difference was on beauty-ideal internalization, which made significant greater positive contributions to attitudes towards cosmetic surgery among Chinese women in China relative to Dutch women. Chinese women seem to live under greater sociocultural pressures to internalize beauty ideal and to conform to social standards [22, 108]. Chinese society is considered more collectivistic and male-oriented; as advocated by Confucius values, women belong to or should subject to the men in their family and social life, and women’s worth is valued in relation to men [22, 23]. In relation to that, women in China are predominantly evaluated based on their appearance instead of personal abilities and qualities in workplace and mate selection [46, 58], particularly under the social force of beauty economy [25]; meanwhile, they are being constantly exposed to idealized beauty and cosmetic surgery information in their daily life [58]. Taken these sociocultural characteristics together, Chinese women seem to be greatly objectified and very encouraged to internalize beauty ideals and subsequently, think of cosmetic surgery as a means to achieve these ideals. In contrast, all of these characteristics may be less salient among Dutch women, in a more individualistic society where ranks also relatively well among countries in gender equity [32, 33].

The other cross-cultural difference was on body appreciation, which could bring significantly greater increase in favorable attitudes towards cosmetic surgery among Chinese women in China compared to Dutch women. As mentioned previously, body appreciation may be conceptualized differently in Chinese and Western cultures. Of note, though body appreciation was a significant predictor also among Chinese women in the Netherlands, finding no difference between them and Dutch women might signify that living in Dutch culture had, somewhat, re-conceptualized body appreciation for them. Future research could longitudinally explore changes in favorable attitudes towards cosmetic surgery and body appreciation among Chinese women who come to the Netherlands.

### Limitations

The following limitations to this study must be mentioned. First, not all potential correlates of attitudes towards cosmetic surgery could be investigated in this study, which leaves room for future investigations. Second, though this study could provide a quantitative cross-cultural understanding about attitudes towards cosmetic surgery, future studies are recommended to explore the qualitative meaning underlying our results. As aforementioned, Wu et al.’s [58] study has provided some qualitative understandings of Chinese and Dutch women’s views of cosmetic surgery, which also helped to substantiate some of our qualitative findings. However, their study was not able to tap into all potential psychosocial perspectives in relation to cosmetic surgery consideration, which could be addressed by future research. Also, their qualitative exploration did not include a group of Chinese women living in the Netherlands or a group of Dutch women living in China, as what our current study did. Further qualitative study could consider including a group of participants living abroad to investigate the role of culture in cosmetic surgery consideration in a richer and exploratory manner. Additionally, participants needed to have internet access to take part in the online survey, and needed to be literate to read the survey questions. Future research should investigate whether the present findings apply to women with more varied economic backgrounds and levels of literacy, making adjustments to the study design and materials as needed. Further, a paper published after our data collection argues that the back-translation technique, which we have employed in this study, has some limitations (see [118] for details), even though our translations were carried out with caution and the reliabilities for the translated measurements were supported. Future studies should consider employing other translation techniques [118]. Moreover, Chinese women completed the questionnaires in Chinese, their mother tongue, but Dutch participants completed the questionnaires in English, their second language. Among countries where English is not the first language, the Netherlands ranks the highest in Europe and worldwide for English proficiency [119] and it is estimated that 90 to 93% of the population are proficient in English [120]. As such, it is common for research in the Netherlands to be conducted in English. Using English measures also enabled us to use the original versions of the questionnaires, when no official Dutch translations would have been available for some of them. Nevertheless, future research among Dutch people could include Dutch translations of the questionnaires, to see whether the present results are confirmed. Finally, due to the fact that Chinese people usually come to the Netherlands for particular purposes and, often, for higher education, the majority of the Chinese participants we recruited in the Netherlands consisted of women with Master or PhD degrees. Though more and more findings show no relationship between education and cosmetic surgery consideration, future studies are encouraged to replicate our study among Chinese women in the Netherlands with more varied educational backgrounds.

### Implication of the findings

Despite the limitations, our current findings add substantially to our understanding of women’s attitudes towards cosmetic surgery from a cross-cultural perspective and have practical and research significance. First, it is interesting that Chinese women living in the Netherlands scored higher on appreciating their bodies and body functionality compared to those in China; however, what might help to explain this finding remains unclear, particularly from our current quantitative data. Hence, researchers are encouraged to conduct qualitative and quantitative research to gain a more fine-grained understanding of how and when Chinese women may develop a more positive body image when living abroad. Second, to the best of our knowledge, there is no well-developed psychosocial intervention for reducing cosmetic surgery consideration documented in the literature. The importance of beauty-ideal internalization in cosmetic surgery consideration has been supported in the literature as well as our current study. Following this line of thought, a dissonance-based intervention, which produced promising effectiveness in improving body image and preventing eating disorders (e.g., [121–124]), might be adaptable and effective for reducing cosmetic surgery consideration; this could be investigated in future research. Meanwhile, future qualitative study could consider acquiring more up-to-date understanding of the meaning of (having) idealized beauty for women, to see whether there is anything beneath their pursuit of beauty. Moreover, valuing materialistic success seems to be a strong motivation for Chinese and Dutch women to undergo cosmetic surgery. Literature has supported that the importance of having materialistic possessions and the relationship between beauty and materialistically satisfactory life are heavily depicted in social media with the development of consumerism (e.g., [45]). Thence, it is important to help women to develop critical thinking ability and to recognize what they truly value and want to pursue in their lives. This is in line with the proposition that body image interventions should go beyond only targeting the body (image) itself and guide women to see their lives with a bird’s eye view, to foster an overall sense of empowerment (e.g., [125, 126]). Last but not least, our findings have quantitatively substantiated Wu et al.’s [58] qualitative finding which implied a positive role of body appreciation in Chinese women’s cosmetic surgery consideration. Their finding suggested that Chinese women might conceptualize the term “body appreciation” differently compared with its formal definition in the Western literature [71], by interpreting body appreciation as self-focused attention, the pursuit of self-perfection, and appearance investment, as well as overlooking the element of unconditional acceptance [58]. Further, Wu et al.’s [58] participants described how Chinese culture and parenting stress the importance of criticism in perfecting the self and the next generation, which is consistent with the literature concerning Confucianism that advocates self-cultivation, self-improvement, and children needing to honor their families by behaving perfectly in all aspects of life (e.g., [115, 127]). Taken together, our finding with the relevant literature all underline the importance of acquiring a more fine-grained understanding of body appreciation, particularly its relationship with body image, in Chinese culture by conducting more qualitative and quantitative research.

## Conclusion

This study has helped to shed light on women’s attitudes towards cosmetic surgery from a cross-cultural viewpoint, and from multiple perspectives. The findings revealed several noteworthy similarities and differences in Chinese and Dutch women’s levels of favorable attitudes towards cosmetic surgery and its potential correlates. The knowledge gained from this study could help to develop culturally-sensitive psychosocial interventions that aim to promote a positive body image, and to assist women who are considering undergoing cosmetic surgery in reflecting upon their real needs beneath such consideration and in evaluating whether undergoing cosmetic surgery is necessary to achieve them. We hope that the present findings inspire future research in this important field, from both quantitative and qualitative perspectives.
